# Influence of public health and infection control interventions during the severe acute respiratory syndrome coronavirus 2 pandemic on the in-hospital epidemiology of pathogens: in hospital versus community circulating pathogens

**DOI:** 10.1186/s13756-022-01182-z

**Published:** 2022-11-11

**Authors:** Laura Dapper, Aline Dick, Claudia Nonnenmacher-Winter, Frank Günther

**Affiliations:** Division of Infection Control and Hospital Epidemiology, Philipps-Universität Marburg, Baldingerstrasse, 35043 Marburg, Germany

## Abstract

**Background:**

The first detection of severe acute respiratory syndrome coronavirus 2 (SARS-CoV-2) in Germany was reported in early February 2020. In addition, extensive control measures on the coronavirus disease 2019 (COVID-19) pandemic have been placed in Germany since March 2020. These include contact and travel restrictions, distance rules, mandatory wearing of face masks and respirators, cancellation of mass events, closures of day-care centers, schools, restaurants and shops, isolation measures, and intensified infection control measures in medical and long-term care facilities. Changes in demand or access to health care services and intensified control measures can lead to changes in transmission dynamics and imply effects on the overall occurrence of infectious diseases in hospitals.

**Methods:**

To analyze the impact of infection control measures implemented in public on infectious diseases in hospitals, surveillance data from Marburg University Hospital were analyzed retrospectively. The analysis was conducted from January 2019 to June 2021, referred to hospital occupancy and mobility data in the county of Marburg-Biedenkopf, and correlated to control measures in hospitals and the general population.

**Results:**

The COVID-19 pandemic and associated measures immediately impacted the occurrence of infectious diseases at the Marburg University Hospital. Significant changes were detected for virus-associated respiratory and gastrointestinal diseases. The massive drop in norovirus infections was significantly affected by the onset of the pandemic (*P* = 0.028). Similar effects were observed for rotavirus (up to − 89%), respiratory syncytial virus (up to − 98%), and adenovirus infections (up to − 90%). The decrease in gastrointestinal and respiratory virus detection rates was significantly affected by the decline in mobility (*P* < 0.05). Of note, since April 2020, there have been no detected influenza cases. Furthermore, *Clostridioides* difficile-related infections declined after late 2020 (− 44%). In contrast, no significant changes were detected in the prevalence of susceptible and drug-resistant bacterial pathogens. In particular, the detection rates of methicillin-resistant *Staphylococcus aureus* isolates or multidrug resistant (MDR) and extended drug resistant (XDR) bacteria remained constant, although the consumption of hand disinfectants and protective equipment increased.

**Conclusions:**

The COVID-19 pandemic and associated public health measures had a significant impact on infectious diseases and the detection of pathogens at the Marburg University Hospital. Significant changes were observed for community transmissible infections, while no such effects on pathogens primarily associated with nosocomial transmission could be detected.

**Supplementary Information:**

The online version contains supplementary material available at 10.1186/s13756-022-01182-z.

## Introduction

The coronavirus disease 2019 (COVID-19) pandemic describes the worldwide outbreak of infections with *severe acute respiratory syndrome coronavirus 2* (SARS-CoV-2), which constitutes the most severe pandemic of the twenty-first century [1]. At the beginning of the pandemic in December 2019, a considerable number of cases of pneumonia caused by an unknown infectious agent were detected in Wuhan (China) [2]. The fast spread of the unexplored infectious disease in China and the rest of the world led to the classification as *Public Health Emergency of International Concern* (PHEIC) and a pandemic by the World Health Organization (WHO) [3, 4].

The pandemic poses a great threat to global health and has massively impaired social life, mobility [5], public health, and economic systems [6]. Only using nonpharmacological intervention (NPI) were most of the national health systems able to respond to the new threat. Numerous research studies focusing on measures of physical distancing have concluded that this is the most effective intervention [7]. The effectiveness of the measures is based on studies confirming that the transmission of the virus mainly occurs by the interaction between individuals [8]. Based on these scientific results, many countries have implemented prevention and control measures to protect vulnerable populations from infection, that is, older adults and those with comorbidities. Most of the national response strategies include the development of measures in public health and infection prevention, various levels of contact tracing, and preparing health systems for a wave of severely ill patients.

The first detection of SARS-CoV-2 in Germany was reported in early February 2020. Extensive control measures on the COVID-19 pandemic have been placed in Germany since the beginning of March 2020. These include contact and travel restrictions, distance rules, mandatory wearing of facemasks and respirators, cancellation of mass events, closures of day-care centers, schools, restaurants and shops, isolation measures, and intensified infection control measures in medical and long-term care facilities. Changes in demand or access to health care services and intensified control measures can lead to changes in transmission dynamics and imply effects on the overall occurrence of infectious diseases in hospitals.

The actual effects of the abovementioned containment strategies on the occurrence of other viral infections and pathogen detection are largely unexplored; thus, further research is required. Nevertheless, by analyzing the effects of newly implemented preventive measures, lessons can be drawn for future outbreak situations and a general reduction of the disease rate. The increasing prevalence of antibiotic-resistant pathogens presents one of the biggest problems in public health services. Furthermore, the occurrence of endemic infectious diseases causes a high disease rate that can only be considerably reduced by effective infection prevention efforts.

Aiming to analyze the influence of the COVID-19 pandemic and the implemented prevention and control measures on other infectious diseases and pathogen detection, the laboratory detections of Marburg University Hospital were analyzed retrospectively between 2019 and 2021. The quarterly detection numbers were set into temporal context due to the COVID-19 pandemic implementing social and clinic in-house NPI measures and correlated with the intensification of established hygiene measures and the change in contact and mobility behavior.

## Methods

In this study, pathogens detection was analyzed retrospectively at the Marburg University Hospital from January 1, 2019 to June 30, 2021. The University Medical Center of Giessen and Marburg (UKGM) is Germany’s third-largest university medical center, where approximately 436,000 patients are being treated each year. Over the entire study period, normal care was largely unaffected in the inpatient area. Outpatient care was partially restricted or perceived to a reduced extent. The analysis included norovirus (104 cases), influenza (272 cases), respiratory syncytial virus (RSV) (102 cases), adenovirus (65 cases), rotavirus (81 cases), *Clostridioides difficile* infection (CDI) (353 cases), methicillin-resistant *Staphylococcus aureus* (MRSA) (430 cases) and multidrug resistance (MDR) (921 cases) and extended drug resistance (XDR) (74 cases) detections within the abovementioned observation period based on the clinically relevant material. The results were collected using standard microbiological and virological methods (Additional file 1). The corresponding diagnostics were performed in the laboratories of the department of hospital hygiene, and medical microbiology and virology of UKGM Marburg. To compensate for possible multiple submissions, only one result per patient case (hospital stay) was included in the analysis. During the investigation period, neither adjustments were made to the screening algorithms or diagnostics, nor were any screening measures carried out as part of outbreak investigations. The detection rates should therefore be comparable for the period before and during the pandemics. For the statistical and graphic analysis, Statistical Package for the Social Science (SPSS) program (IBM, Armonk, USA) was used. Using two-factor variance analysis, the extent to which the COVID-19 pandemic and respective quarter influenced the detection rates were examined (H_0_: neither the factor quarter [Q1–Q4] nor the factor COVID-19 [0 = no COVID; 1 = COVID] led to a significant change in the detection rates). The basis of a 95% confidence interval was the significance level of *P* = 0.05. The adjustment of the α-level of the post hoc tests was performed according to Bonferroni. The effect size was determined using the partial eta-square (${\eta }_{p}^{2}$). Furthermore, the extent to which the changes in the quarterly detection rates during the pandemic correlated with changes in mobility in Germany and the county of Marburg-Biedenkopf was analyzed. Here, the mobility data analyzed by the COVID-19 mobility project of the Humboldt University Berlin in cooperation with the Robert-Koch-Institute were included [9]. The correlation coefficient of the bivariate correlation was calculated according to Spearman-Rho with a significance level of *P* = 0.05 in SPSS. The occurrences during the COVID-19 pandemic and the accompanying prevention measures, were displayed on a timeline. In-hospital measures were shown based on the use of disinfectants (data not shown) and antibiotics and the demand for personal protective equipment.

## Results

**Fig. 1:**
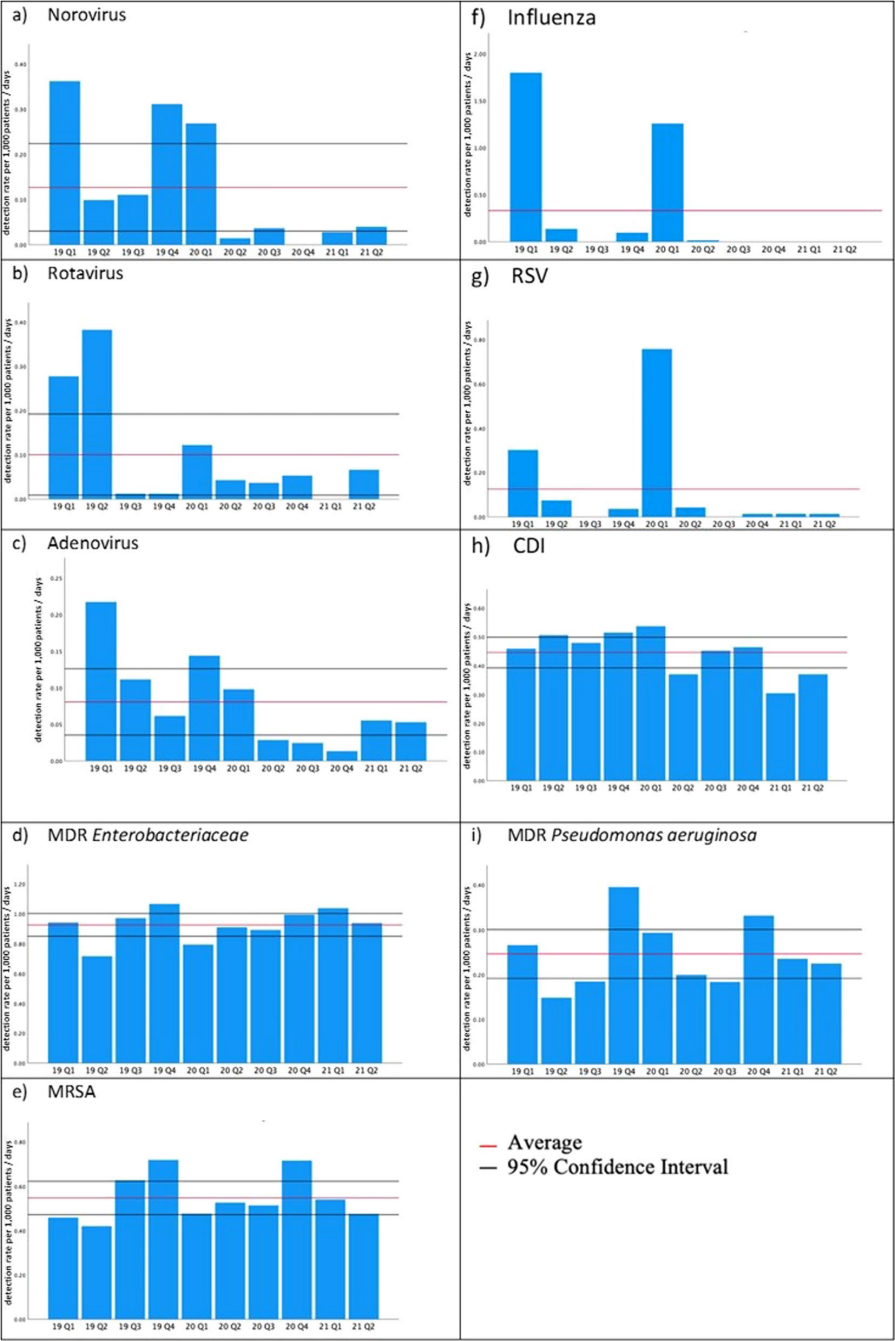
Rates of infectious diseases and the detection of pathogens at the Marburg University Hospital (per 1,000 patient/days) **a** Norovirus, **b** Rotavirus, **c** Adenovirus, **d** MDR Enterobacteriaceae, **e** MRSA, **f** Influenza, **g** RSV, **h** CDI, **i** MDR *P*. *aeruginosa*

**Fig. 2:**
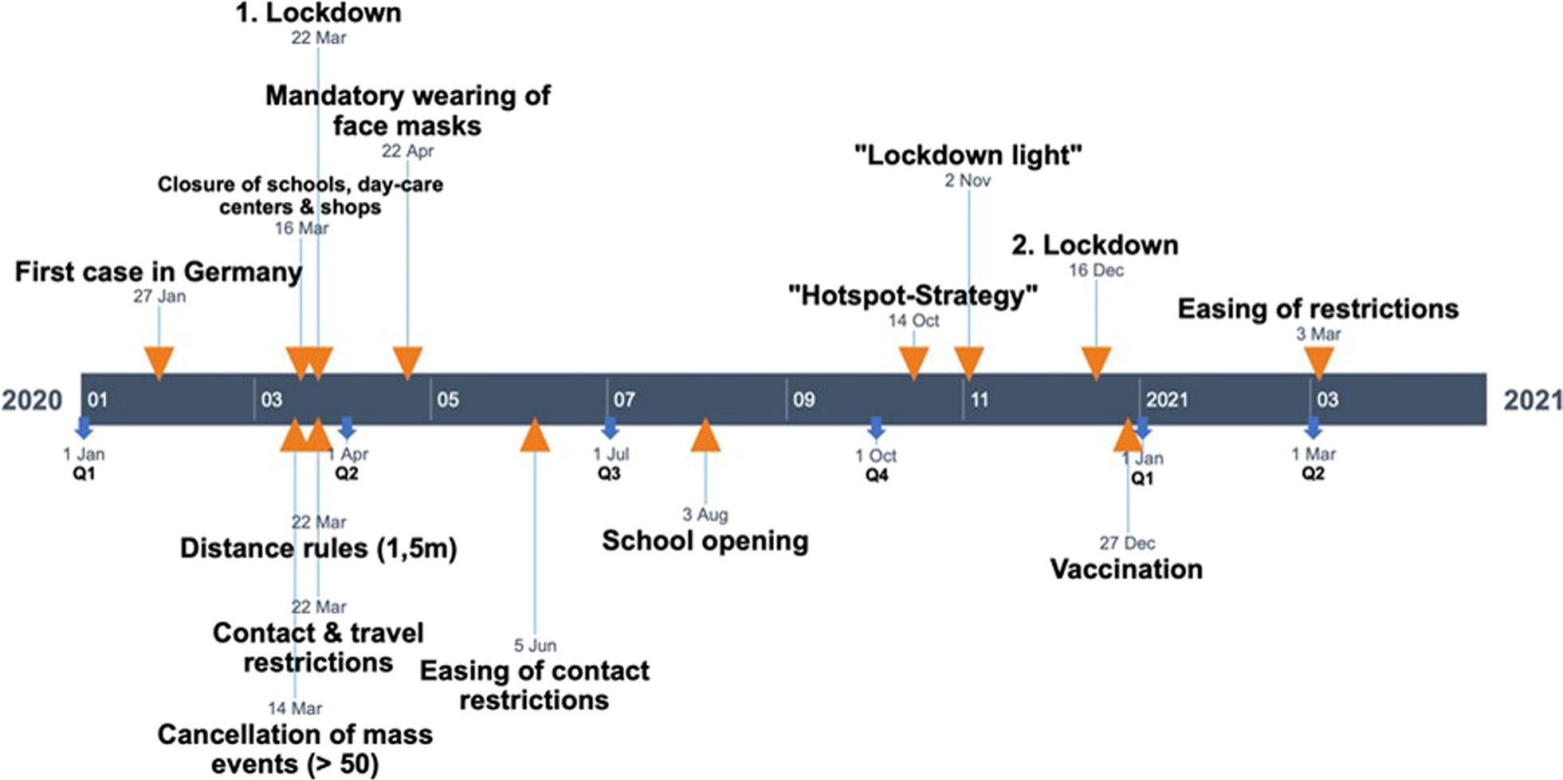
Control measures and events associated with the COVID-19 pandemic in Germany

The COVID-19 pandemic and associated prevention and control measures immediately impacted the occurrence of infectious diseases at the Marburg University Hospital (Fig. 1). Significant changes were detected for virus-associated respiratory and gastrointestinal diseases. The massive drop in norovirus infections was significantly affected by the onset of the pandemic (*P* = 0.028). Similar effects were observed for rotavirus (up to − 89%), RSV (up to − 98%), and adenovirus infections (up to 90%). The decrease in gastrointestinal and respiratory virus detection rates was significantly affected by the decline in mobility (*P* < 0.05) due to travel and contact restrictions. Travel restrictions were part of the extensive control measures that have been placed in Germany since the beginning of March 2020 (Fig. 2). Of note, since April 2020 until June 2021, there have been no detected influenza cases. Furthermore, *C. difficile*-related infections declined after late 2020 (− 44%). In contrast, no significant changes were detected in the prevalence of susceptible and drug-resistant bacterial pathogens. In particular, the detection rates of MRSA isolates or MDR and XDR bacteria remained constant and no statistically significant changes in the detection rates of MDRO compared between the period before and during the pandemic could be detected.

## Discussion

The present study shows that NPI measures implemented during the COVID-19 pandemic to contain the SARS-CoV-2 virus also considerably influences the occurrence of other clinically relevant pathogens. Furthermore, the changes in demand for healthcare services and implement prevention and control measures led to a significant change in transmission dynamics.

The analysis of the laboratory detections of Marburg University Hospital shows that due to the implementation of the NPI measures, gastroenterological and respiratory viral infections were drastically reduced. The number of norovirus infections dropped dramatically, which became statistically significant after the pandemic emerged (*P* = 0.028). The same applies to rotavirus (up to 89%) and adenovirus enteritis, showing significantly lower detection rates. Seasonal clusters, which usually occur with norovirus and rotavirus infections, completely failed to appear after implementing social intervention. Similar data were reported by Grochowska et al., who focused on changes in the occurrence of transmissible viral infections in children [10]. Their microbiological analysis (until April 2021) documented 87% fewer rotavirus infections and 47% norovirus infections during the pandemic. The drastic drop in the detection rates of gastroenteritis viruses at the Marburg University Medical Center was significantly correlated with the decline in mobility in the county (*P* < 0.05) caused by social and travel restrictions, working at home, and the extensive limitations of public events. The abrupt interruption of common chains of infections may be caused by limiting private and public contact. Especially in the winter months, during which norovirus and rotavirus predominantly circulate, social contacts between more than 10 people rarely occur. The contact monitor at the Humboldt University Berlin clearly shows this. Based on the assumption that transmissions occur if people have social contacts, a group of researchers analyzed the GPS data of mobile phones in Germany after the social restriction was implemented; thus, it displayed the course of the pandemic with regard to social contacts [11]. During the first lockdown (beginning March 22, 2020), a massive drop in social contacts of approximately 50% was observed compared with the pre-pandemic level (19 compared with 9 contacts per person per day). Furthermore, the discontinuation of mass catering at restaurants or cultural events plays a significant role in reducing the risk of transmission by contaminated food. The temporary closing of schools and nurseries also led to the absence of one of the predominant locations of transmission of gastroenteritis in children. The enhanced awareness of the population regarding hand hygiene measures may also represent an important reason for the significant reduction in viral gastroenteritis.

Additionally, a significant decline was observed in respiratory viral infections. There was no increase in influenza cases in the winter of 2020/2021. The usual pre-pandemic seasonal occurrence of RSV detection in the first quarter of 2021 (-98% compared with Q1 2020) did not occur. These changes in detection rates were also significantly correlated (*P* < 0.05) with the changes in mobility. Apart from the aforementioned causes, the general mask mandate, social distancing, and proactive self-isolation in the case of respiratory symptoms due to social expectancy were mainly responsible for the changing dynamics of transmission and the nonoccurrence of detections. During the influenza A (H1N1) pandemic in 2009, national and international public health authorities recommended wearing masks to reduce the risk of transmission. However, owing to the insufficient availability of data, no actual evidence regarding the protective effect of this barrier precaution can be delivered [12]. Nevertheless, the latest studies conducted during the COVID-19 pandemic showed that surgical masks could reduce the transmission of human coronaviruses and influenza viruses by people with symptoms [13]. A meta-analysis by Liang et al., which included 21 studies, confirmed the significant protective effect of masks against influenza virus (odds ratio [OR] = 0.55), SARS (OR = 0.6), and SARS-CoV-2 (OR = 0.04) [14]. Thus, wearing a mask during the SARS-CoV-2 pandemic seems to be one of the main reasons for the detection rates of influenza and RSV.

The implementation of social distancing contributes to the massive reduction in respiratory viral infections. In a meta-analysis including 172 observational studies, Chu et al. stated that the risk of transmission of SARS-CoV-2 viruses could be significantly reduced by a physical distance of 1 m or more (n = 10 736, pooled adjusted OR [aOR] 0.18, 95% CI 0.09 to 0.38) [15]. The level of protection increased with increasing distance (change in relative risk [RR] 2.02 per m; *P* = 0.041). Infections caused by *C. difficile* were reduced by up to 44% (Q1 2021) during the pandemic. This was confirmed by Bentivegna et al., who observed a significant decline in *C. difficile* cases after the outbreak of the COVID-19 pandemic. Their analysis covered the period from March to June 2020; however, they excluded intensive care units and children’s medical units [16]. The reduction in detection at the Marburg University Hospital was significantly correlated with the change in nationwide mobility. The reason for this seems to be particularly the reduced demand for outpatient services, which caused a reduction in antibiotic prescriptions; thus, led to a reduction in CDI rates. A change in risk factors for the development of an antibiotic therapy-related CDI in the hospital is unlikely from our perspective due to the unchanged application of antibiotics and the efforts of antibiotic stewardship and suggests changes in outpatient medical and public areas. However, a reduction in *C. difficile* cases due to intensified hygiene measures in hospitals and other medical centers were also suggested by Sipos et al. [17] and Bentivegna et al. [16]. For example, intensified hygiene measures indicated increased cleaning and disinfection intervals and use of personal protective equipment, pose a relevant factor only if the level of standard hygiene was low before the pandemic. Regional and national differences with respect to hygiene requirements in hospitals and their implementation are reasons for the diminished comparability of the initial situation. An increase in already established standard hygiene measures could not be observed at the Marburg University Hospital, except for the use of hand disinfectants, which, according to other studies, does not correlate with the incidence of CDI [18].

At the Marburg University Hospital, enhanced infection prevention and control (IPC) did not lead to reduced detection of antibiotic-resistant bacteria, as has been suggested by various research groups. No changes were observed with respect to the MRSA, MDR, and XDR rates. Wee et al. observed a reduction in MRSA acquisitions from 11.7 cases per 10,000 hospitalization days to 6.4 cases (*P* < 0.05). However, with respect to the acquisition of carbapenem-resistant *Enterobacteriaceae* (CRE), no noteworthy changes could be discovered during the observation period. Baccolini et al. observed an increase in the incidence of patients with healthcare-associated infections (HAIs), especially device related HAIs, which may be attributed to a collateral damage to long-established infection control measures by focusing resources to primarily mitigate the spread of SARS-CoV-2 [19].

The decline in MRSA detection rates during the pandemic discovered by several studies can be evaluated as a result of intensified hygiene measures [20, 21]. Similar effects were not observed at the Marburg University Hospital despite the significantly increased use of disinfectants. A possible reason for this could be the point in time or indication for hand disinfection, for which increased consumption was recorded during the pandemic. According to the observation of hand disinfection by Israel et al., personal protection and patient protection moments must be differentiated [22]. Thus, after contact with the patient, disinfection measures serve to protect the patient. Therefore, it can be assumed that increased use of disinfectants first helped protect the staff and did not influence the detection rates of the patients.

Furthermore, it seems clear that the pre-pandemic level of measures for infection prevention is the predominant factor for effect displayed on MDR and XDR rates due to intensified IPC measures during the COVID-19 pandemic. High-income countries already have high standards of antibiotic stewardship and infection prevention; thus, the enhancements of resistance situations and detection rates are perceived as smaller. The same applies to the effect of intensification of hygiene measures, such as hand disinfection. At the Marburg University Hospital, compliance with hand disinfection and basic hygiene standards had already been high. Therefore, the application of antibiotics and the efforts of antibiotic stewardship did not change during the pandemic. This seems to be one possible explanation for the consistent MDR rates. However, limiting factors of our study is that it was conducted with a single healthcare provider and only in one distinct geographic region with a defined patient community; hence, the findings may not be fully generalizable to other settings.

## Conclusion

The COVID-19 pandemic and associated public health measures have had a significant impact on the occurrence of infectious diseases and the detection of pathogens in hospitals. Significant changes were observed for community-transmissible infections, while no such effects on pathogens primarily associated with nosocomial transmission could be detected. In addition, the detection rates of gastroenterological and respiratory viral infections correlated significantly with the implemented public health measures and the changes in mobility; thus, locating the place and route of transmission in the general public rather than in hospitals. On the other hand, MDR bacteria circulate primarily in hospitals and are exposed to the same selection pressure during the pandemic. Therefore, the implemented public health measures and changes in mobility while maintaining standard hygiene measures had no impact on the prevalence of MDR.

## Supplementary Information

**Additional file 1. Supplement 1**. Pathogens and respective test procedures applied in the study.

## Data Availability

The datasets used and/or analysed during the current study are available from the corresponding author on reasonable request.

## Abbreviations

SARS-CoV-2: Severe acute respiratory syndrome coronavirus 2
COVID-19: Coronavirus disease 2019
WHO: World Health Organization
PHEIC: Public Health Emergency of International Concern
NPI: Nonpharmacological intervention
UKGM: University Medical Center of Giessen and Marburg
RSV: Respiratory syncytial virus
CDI: Clostridioides difficile infection
MRSA: Methicillin-resistant Staphylococcus aureus
SPSS: Statistical Package for the Social Science
MDR: Multidrug resistance
XDR: Extended drug resistance
