# Supplementary material for: Influence of public health and infection control interventions during the severe acute respiratory syndrome coronavirus 2 pandemic on the in-hospital epidemiology of pathogens: in hospital versus community circulating pathogens
Source: Antimicrob Resist Infect Control. 2022 Nov 11;11:140. doi: 10.1186/s13756-022-01182-z (PMC9651880; doi:10.1186/s13756-022-01182-z)
Supplement: Supplementary file 1 — Additional file 1. Supplement 1. Pathogens and respective test procedures applied in the study. [file 13756_2022_1182_MOESM1_ESM.pdf]

Supplement 1

Pathogens and respective test procedures applied in the study

	indication	specimen/ test procedure	Evaluation
**Norovirus**	symptomatic disease	stool sample<br><br>PCR, antigen detection	Quarterly detection rate: (detections/1000 patient days)<br><br>1 detection per patient and stay
**Rotavirus**	symptomatic disease	stool sample<br><br>PCR, antigen detection	Quarterly detection rate: (detections/1000 patient days)<br><br>1 detection per patient and stay
**Adenovirus**	symptomatic disease	stool sample<br><br>PCR, antigen detection	Quarterly detection rate: (detections/1000 patient days)<br><br>1 detection per patient and stay
**Influenza**	symptomatic disease	respiratory specimen<br><br>PCR, antigen detection	Quarterly detection rate: (detections/1000 patient days)<br><br>1 detection per patient and stay
**RSV**	symptomatic disease	respiratory specimen<br><br>PCR, antigen detection	Quarterly detection rate: (detections/1000 patient days)<br><br>1 detection per patient and stay
**CDI**	symptomatic disease	stool sample<br><br>ELISA, culture	Quarterly detection rate: (detections/1000 patient days)<br><br>1 detection per patient and stay
**MRSA**	Screening, symptomatic disease	nasal swab, clinical specimen<br><br>PCR, culture	Quarterly detection rate: (detections/1000 patient days)<br><br>1 detection per patient and stay
**MRGN**	Screening, symptomatic disease	rectal swab, clinical specimen<br><br>PCR, Kultur	Quarterly detection rate: (detections/1000 patient days)<br><br>1 detection per patient and stay
